# A quantitative taxonomy of human hand grasps

**DOI:** 10.1186/s12984-019-0488-x

**Published:** 2019-02-15

**Authors:** Francesca Stival, Stefano Michieletto, Matteo Cognolato, Enrico Pagello, Henning Müller, Manfredo Atzori

**Affiliations:** 1 Intelligent Autonomous Systems Lab (IAS-Lab), Department of Information Engineering (DEI), University of Padova, Padova, Italy; 2 Information Systems Institute, University of Applied Sciences Western Switzerland (HES-SO), Sierre, Switzerland; 3 Rehabilitation Engineering Laboratory, Department of Health Sciences and Technology, ETH Zurich, Zurich, Switzerland; 4 Now retired from academy, and with EXiMotion Srl, Via Prima Strada, 35, Padova, Italy; 5 University of Geneva, Geneva, Switzerland

**Keywords:** Hierarchical trees, Electromyography, Kinematics, Hand Grasps Taxonomy, Hand Movements, Robotics

## Abstract

**Background:**

A proper modeling of human grasping and of hand movements is fundamental for robotics, prosthetics, physiology and rehabilitation. The taxonomies of hand grasps that have been proposed in scientific literature so far are based on qualitative analyses of the movements and thus they are usually not quantitatively justified.

**Methods:**

This paper presents to the best of our knowledge the first quantitative taxonomy of hand grasps based on biomedical data measurements. The taxonomy is based on electromyography and kinematic data recorded from 40 healthy subjects performing 20 unique hand grasps. For each subject, a set of hierarchical trees are computed for several signal features. Afterwards, the trees are combined, first into modality-specific (i.e. muscular and kinematic) taxonomies of hand grasps and then into a general quantitative taxonomy of hand movements. The modality-specific taxonomies provide similar results despite describing different parameters of hand movements, one being muscular and the other kinematic.

**Results:**

The general taxonomy merges the kinematic and muscular description into a comprehensive hierarchical structure. The obtained results clarify what has been proposed in the literature so far and they partially confirm the qualitative parameters used to create previous taxonomies of hand grasps. According to the results, hand movements can be divided into five movement categories defined based on the overall grasp shape, finger positioning and muscular activation. Part of the results appears qualitatively in accordance with previous results describing kinematic hand grasping synergies.

**Conclusions:**

The taxonomy of hand grasps proposed in this paper clarifies with quantitative measurements what has been proposed in the field on a qualitative basis, thus having a potential impact on several scientific fields.

## Background

In 1989 Cutkosky [1] said that the main goal in the field of rehabilitation robotics was to build a robot capable of deciding autonomously how to pick up and manipulate objects to perform everyday tasks just like humans do. However, the human hand can perform an almost infinite number of movements. Structuring and organizing the hand grasps into a hierarchical taxonomy can be useful to better understand how the hands interact with different objects as well as to advance and evaluate devices that try to imitate them.

A taxonomy of hand movements is important for several scientific fields, including robotics, prosthetics, physiology and rehabilitation. In robotics, it can be useful to compare the functionality of robotic hands with real human hands. In prosthetics, very advanced myoelectric hands have been developed from a mechanical point of view but they are usually not well accepted by amputees [2–4]. A taxonomy of hand grasps can foster the development of prosthetic hands that perform movements corresponding to the taxonomic groups that are mostly useful in real life situations. In physiology, a comprehensive quantitative comparison of hand grasps may create a link between hand synergies [5] and real life needs. In rehabilitation, a proper taxonomy of human grasps allows prioritizing the hand functionalities that need to be restored with the highest priority. Santello et al. [5] proposed an early approach into this direction by applying Principal Component Analysis (PCA) to digit joint angles during a significant set of hand postures. Their work, as many others that followed it [6–11], took inspiration from grasp taxonomies in order to properly select the set of hand movements.

Several attempts to build a complete taxonomy of hand grasps were published in the scientific literature during the last 30 years. However, all of the presented taxonomies were based on qualitative approaches and qualitative justifications. Most of the taxonomies of hand movements include a division between power and precision tasks. This idea was originally proposed by Napier et al. in 1956 [12] and influenced most authors afterwards (e.g. [13–16]). Cutkosky [16] organized 16 hand grasps into a hierarchical tree according to the adaptability required by small-batch tasks. The grasps were characterized using several qualitative measures (such as compliance, connectivity, grasp isotropy, resistance and other parameters) and they were split into power and precision tasks. Feix et al. [17] compared several previous taxonomies of hand grasps and created a taxonomy of hand grasps that they called the *GRASP* taxonomy. This taxonomy is organized in a matrix, with the grasps divided into several columns and in two rows according to four main parameters including power type, opposition type, position of the thumb and virtual finger assignments. Starting from Feix’s work, Wolf et al. [18] considered composed tasks in order to evaluate the micro-gestures that can be performed alongside the main grasp. More recently, Bullock et al. [19] decomposed manipulation tasks into simpler movements with an object-centric, environment-centric and hand-centric perspective. This taxonomy provides a structured way to classify 15 simple movements, where basic movements can be composed in order to build more complex movements.

Qualitative methods can provide useful perspectives of nature. However, quantitative measurements are strongly related to the scientific method and to the concept of science itself. Quantitative methods provide practical control over the subject studied, they make possible a formulation of principles that are capable of unambiguous confirmation or refutation (depending on experiments and measurements) possible and therefore very few investigations can be carried out without them [20].

A quantitative taxonomy of hand movements can therefore reduce ambiguity in the field, but it requires the measurement of specific biomedical data. Several parameters can be used to quantitatively characterize hand grasps, such as posture, muscular activity and force. Kinematic data are usually measured with two main techniques: visual or wearable systems. Visual systems can be affected by visual occlusion in the recording of hand grasps and the procedure to place the visual markers can be time consuming. Data gloves are a common alternative that is sufficiently precise [21] and extremely easy to record. Thus, they are suitable for studies involving many subjects. The joint angles were previously used as features in order to compare model estimations with real position measurements [22, 23]. In the comparison of movements, synthesis functions are often applied to represent the entire motion with fewer data. For instance, Finger Aperture Indexes (FAIs) were used to represent long finger opening starting from joint angles collected by a Motion Capture (MoCap) system composed of nine infrared cameras and 17 retro-reflective hemispheric markers [24]. Normalized geometric distances were used as features for representing hand gestures [25].

Muscular data can be measured with Surface Electromyography (sEMG). The sEMG signal can be modeled as a superimposition of the Motor Unit Action Potentials (MUAPs) of the active Motor Units (MUs) [26, 27]. The MU recruitment and firing frequency are the major factors for both Electromyography (EMG) amplitude and force exerted by the muscle [27, 28]. Thus, a qualitative relation between the sEMG signal amplitude and the force exerted by the muscle can be noticed [28]. Signal features based on sEMG signal amplitude can reveal the hand movement patterns based on the sEMG amplitude-force relation in both intact subjects [29] and hand amputees [30]. Muscle activation patterns can differ strongly between intact and transradial amputees, particularly in relation to clinical parameters such as phantom limb sensation intensity, remaining forearm percentage and time since the amputation [30]. This result leads to the fact that signal acquisition controls trained on intact subjects may not be valid for amputees [31]. Other parameters may be interesting but in this work we focus on kinematics and muscular activity because we mainly target the posture of the hands but also due to practical data availability.

This paper presents the first quantitative taxonomy of hand movements. The relative variations between joint bending angles (measured with a data glove) allow a quantitative characterization of the hand movement kinematics. The sEMG signals allow a functional analysis of the muscles involved in each grasp. The taxonomy is organized in a hierarchical structure and it is based on a signal feature extraction procedure that is common in sEMG literature.

The problem of computing phylogenetic trees with a hierarchical structure is common in biology. Phylogenetic trees represent the evolutionary relationships among sets of organisms or groups of organisms, representing the ancient idea of a “Tree of Life”. From a phylogenetic point of view, phylogenetic trees are nothing else than a particular kind of family tree. The tree structure groups organisms with unique and shared characters. The more the considered characters are similar the higher the possibility that two subjects belongs to the same group. The more the characters are diverse, the longer the distance between the individuals. If phylogenetics looks for common genetic characters among a group of organisms, then in our case, we are aiming to detect similar features in a set of grasps. In the former, the same parent node in the tree indicates the presence of a shared ancestor, in the latter being part of the same subtree strongly suggests a common underling behavior. We analyze the movements performed by 40 intact subjects to extract the common underlying patterns that characterize each grasp. We create hierarchical trees for each subject individually and subsequently merge them into supertrees to obtain a generalized taxonomy. The problem of merging phylogenetic trees into a single structure emerges often in biology, where this procedure is commonly used to compute a branching history of species. Many practical approaches have been proposed in the literature for fusing information from several trees or for mixing trees in a common structure. Kluge proposed the “total evidence” approach, which claims that the phylogenetic analysis should consider all the available information [32]. The examples proposed in the physiological context consider the fusion of different trees to build legit representations of all the available information, even on very different data. Hinchliff et al. [33] proposed to assemble trees from several features into a comprehensive global reference taxonomy with an efficient and automated process to reunite all phylogenetic relationships to common lineages (“Tree of Life”). Some recent articles proposed to create hierarchies by means of agnostic autonomous approaches like Deep Neural Networks (DNNs). In [34], authors used a max-pooling approach to shrink the representations of higher layers so that their model can perform hierarchical inference of object parts over full-size image. Farabet et al. [35] proposed a method to automatically represent a scene by starting from a family or a tree of oversegmentations. Szalkai and Grolmusz [36] on the other hand developed a web algorithm able to classify biological sequences into a hierarchical structure. In our specific case, we transferred these concepts to a completely different context but with a similar goal. We aim at computing a global taxonomy of hand movements starting from several highly specific taxonomies. The quantitative approach ensures a repeatable non-subjective perspective of this taxonomy, thus making it a reference for several scientific fields.

## Methods

This section describes how kinematic and sEMG data were recorded and analyzed to create a quantitative taxonomy of hand movements. The data analysis procedure can be summarized as data acquisition (“Data acquisition” subsection), signal feature extraction (“sEMG and data glove signal processing” subsections), creation of the hierarchical trees (“Hierarchical trees” subsection) and fusion of the trees into super-trees(“Computation of the muscular, kinematic and general quantitative taxonomies: hierarchical super-trees” subsection), a procedure coming from genetics studies and leading to the general quantitative taxonomy of hand movements.

### Data acquisition

The used dataset is the second Ninapro dataset, including 40 intact subjects (28 males, 12 females; 36 right handed, 4 left handed; age 29.9 ± 3.9 years). The Ninapro database^1^ [29, 37], is a publicly available resource aiming at improving the control of robotic hand prostheses. The data comprise 50 hand and wrist movements, including basic motions (e.g. flexion, extension) as well as 20 grasps.

#### Acquisition setup

The acquisition setup includes a data glove and a set of surface electromyographic electrodes with built-in accelerometer. Hand kinematics were measured using a 22-sensor CyberGlove II (CyberGlove Systems LLC 2), providing data proportional to joint angles, sampled at slightly less than 25 Hz. Muscular activity was measured using a Delsys Trigno Wireless system. The sEMG electrodes are double-differential and measure the myoelectric signals at 2 kHz with a baseline noise of less than 750 nV RMS. The sEMG electrodes were placed using the hypo-allergenic Trigno Adhesive Skin Interfaces. Prior to electrode placement the skin was cleaned with isopropyl alcohol.

**Fig. 1:**
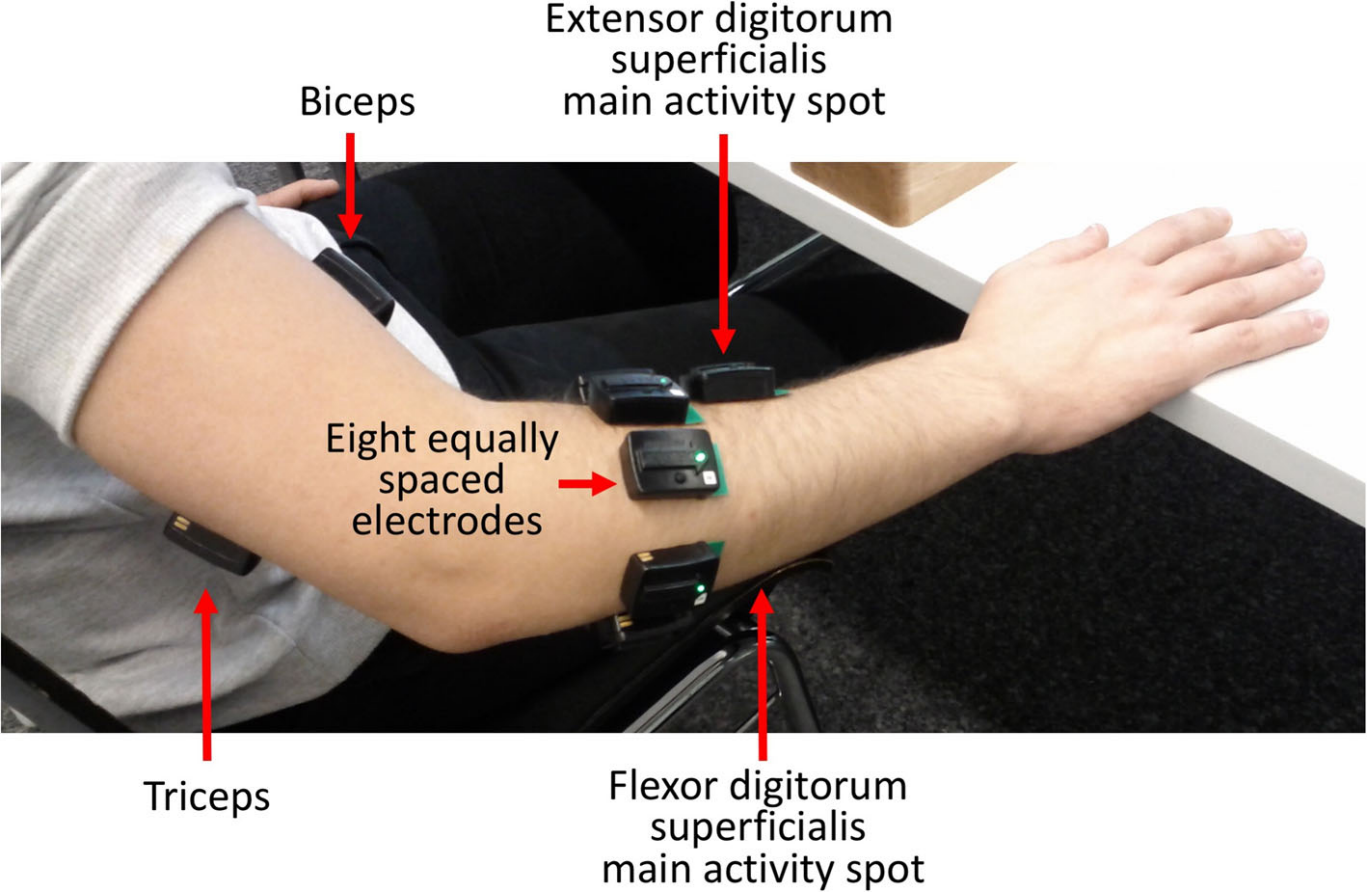
Experimental setup. Acquisition setup and sEMG electrode position

A hand movement is the result of an activation pattern potentially involving several muscles controlling hand and wrist. Therefore, in order to identify the hand movement from the sEMG signal by means of pattern recognition methods, the electrodes were placed around the subject’s forearm combining a precise anatomical positioning strategy [28] with a dense sampling approach [38, 39]. An array of eight sEMG electrodes was applied at the height of the radio-humeral joint. The electrodes were equally spaced, creating an array covering the whole circumference of the forearm. Four electrodes were placed on the main activity spots of four specific muscles: the *flexor digitorum superficialis*, the *extensor digitorum superficialis*, the *biceps brachii* and the *triceps brachii* (Fig. 1). The aforementioned strategy is widely used in the prosthetic field. It was shown that, in terms of pattern recognition accuracy for hand movement identification, the electrode position is not a crucial aspect as long as a sufficient number of channels is provided [40, 41].

#### Acquisition protocol

During the data acquisitions, the subjects were sitting with the arms positioned in a relaxed way on a desktop. A laptop computer was used to show them the videos representing the movements to be performed and to record the data from the sensors. The subjects were asked to synchronously mimic the movements with their right hand. Each subject performed 6 repetitions of 49 movements plus rest. Each movement repetition lasted 5 s, alternated with 3 s of rest. Several precautions were taken in order to encourage a natural and spontaneous execution of the grasp. First, the subjects were instructed to perform the grasp movement as naturally as possible, without lifting the objects or exerting unnatural grip force. The movements were not randomized and the objects to be grasped were positioned as closely as possible to the hand of the subject. The latter also helped in minimizing the time of the reaching and releasing phases. The hand movements were selected from the hand taxonomy, robotics, and rehabilitation literature (e.g., [1, 14, 17, 42]) according to Activity of Daily Living (ADL) requirements. Everyday objects that can easily be found in daily life tasks were used in the experiment.

### sEMG and data glove signal processing

In order to allow the creation of the sEMG based quantitative taxonomy, pre-processing and feature extraction were performed. First, data preprocessing was performed to assure good data quality. This phase included filtering and synchronization. Second, the information of the sEMG signals was made usable by extracting a set of signal features using a moving window technique [43, 44]. Finally, the signal features were used as input data to compute the hand movement taxonomies.

The CyberGlove data were analyzed with a procedure that takes inspiration from window based time series analysis and in particular from the literature in EMG data analysis [29, 43]. The procedure includes synchronization and feature extraction.

#### Filtering

The Delsys electrodes are not shielded against power line interference, so the power line interference was removed using an Hampel filter at 50 Hz [45].

#### Synchronization

A high-resolution timestamp based on the Time Stamp Counter (TSC) of the CPU was assigned to each sample recorded for both the sEMG and joint angle data. The timestamp was used in the post-processing phase to synchronize the devices. To do so, all the modalities were up-sampled at the sampling frequency of the fastest device (2 kHz) using linear-interpolation. This is a well-known technique to increase resolution, avoid aliasing, and reduce noise [46]. Interpolation is particularly useful when the data collected with low frequency (kinematics) is considerably smoother than the data at high frequency (sEMG).

#### Feature extraction

Signal feature extraction was performed applying the method described by Englehart et al. [43]. Each movement repetition was windowed using a 200 ms window, with 100 ms of overlap. As described in scientific literature, diverse signal features highlight different signal properties, leading for instance to varying classification performance (e.g. [29]). In order to make the taxonomy robust to differences between features, a selection of five time domain signal features was extracted on each time window.

The features were chosen according to use in the previous literature and include Root Mean Square (RMS), Mean Absolute Value (MAV), Integrated Absolute Value (IAV), Time Domain Statistics (TD) [44] and Waveform Length (WL) [43, 47–52]. The Time Domain Statistics (TD) are composed of: Mean Absolute Value (MAV), Mean Absolute Value Slope (MAVS), Zero Crossings (ZC), Slope Sign Changes (SSC) and Waveform Length (WL) [44]. Each feature was extracted from each signal *x* on each time window *w* of *T* samples in length.

Root Mean Square (RMS) is arguably one of the most common features to represent sEMG signals. RMS provides a useful measurement of signal amplitude and, under ideal conditions, it has a quasi-linear relationship with the force exerted by a muscle [28]. The RMS feature for a given time window *w* was obtained as

$${RMS}_{w}(x) = \sqrt{\frac{1}{T}\sum\limits_{t=1}^{T}x_{t}^{2}}; $$

where *x*_*t*_ is the *t*^*th*^ sample in the window *w*.

The Mean Absolute Value (MAV) and the Integrated Absolute Value (IAV)^3^ are also popular features in sEMG signal analysis and for a given time window *w* they are defined as [44, 53]:

$${MAV}_{w}(x) = \frac{1}{T}\sum\limits_{t=1}^{T}|x_{t}|\qquad {IAV}_{w}(x) = \sum_{t=1}^{T}|x_{t}| $$

The Mean Absolute Value Slope (MAVS) is defined as the difference between the MAV value of two adjacent time windows, *w* and *w*+1 [44]:

$${MAVS}_{w}(x) = {MAV}_{w+1}(x) - {MAV}_{w}(x) $$

The Zero Crossings (ZC) [44] feature gives an indication about the frequency of the signal by counting how many times the signal crosses zero. The ZC of a signal *x* in a given window *w*, *ZC*_*w*_(*x*), is increased by one if, given two consecutive samples *x*_*t*_ and *x*_*t*+1_, { *x*_*t*_>0 and *x*_*t*+1_<0} or { *x*_*t*_<0 and *x*_*t*+1_>0} and |*x*_*t*_−*x*_*t*+1_|≥ threshold.

Another feature related to the frequency of the signal is the Slope Sign Changes (SSC) [44] which is defined as the number of times the sign of the slope changes. The SSC of a signal *x* in a given window *w*, *SSC*_*w*_(*x*), is incremented if, given three consecutive samples *x*_*t*−1_, *x*_*t*_ and *x*_*t*+1_, { *x*_*t*_>*x*_*t*−1_ and *x*_*t*_>*x*_*t*+1_} or { *x*_*t*_<*x*_*t*−1_ and *x*_*t*_<*x*_*t*+1_} and { |*x*_*t*_−*x*_*t*+1_|≥ threshold or |*x*_*t*_−*x*_*t*−1_|≥ threshold}.

Waveform Length (WL) returns a single parameter providing a measure of the waveform complexity and given a time window *w* it is defined as [44]:

$${WL}_{w}(x) = \sum\limits_{t=2}^{T}|x_{t} - x_{t-1}| $$

### Hierarchical trees

The quantitative taxonomy of hand movements is based on a hierarchical structure in order to highlight dependencies and relationships between the different motions. For each subject, one hierarchical tree was computed for each modality-feature combination, thus leading to five hierarchical trees for the EMG data and five trees for the kinematic data. We adopted this approach instead of building only one large hierarchical tree containing all the subjects in order to achieve a higher control of intermediate results and to be able to check the similarity across subjects. For each subject modality-feature combination, the hierarchical trees were computed by performing one-way Multivariate Analysis of Variance (MANOVA) [54] between the hand movements. This procedure allows us to test our hypothesis for all the movements at once to measure how much a grasp is correlated to the others. Therefore, MANOVA can provide a measure of similarity between the grasps that were considered in the study. Moreover, MANOVA is a standard, well accepted means of performing multivariate analysis. The signal features were grouped by movement and the means of the collected measures were compared by computing the Mahalanobis distance [55]. It is multi-dimensional, unitless, and scale-invariant. The Mahalanobis distance takes into account the correlations coming from the MANOVA procedure to measure how distant a specific movement is from the distribution (the whole set of grasps) in terms of standard deviations. The distances between the movements were then used as a basis to build the dendrograms. The dendrograms were initially represented as binary trees composed of clusters of two movements combined depending on the distance. We followed a hierarchical agglomerative clustering or bottom-up approach. By doing so, we treated each movement as a singleton cluster and then agglomerate pairs of clusters until all clusters are merged into a unique tree containing all grasps. The initial set of grasps was based on previous knowledge so it almost naturally implied the use of hierarchical agglomerative clustering. On the contrary using a divisive (or top-down) approach could have lead us to a different final number of grasps not corresponding to the initial set that was available. Subsequently, the dendrograms were converted into phylogenetic trees that are unordered rooted trees with unweighted edges, with the characteristic of having all the leaves at the same distance from the root. Part of the information contained in the dendrograms is lost when using unweighted edges. This is due to a limitations of the merging algorithm (“Computation of the muscular, kinematic and general quantitative taxonomies: hierarchical super-trees” subsection) that is not currently able to manage such information. Using a weighted structure may provide more accurate results than the current work, thus we are working on an improved version of the merging procedure. Nevertheless, an approach based on unweighted trees is important to have a proper understanding of the general methodology since this is the first attempt to obtain a quantitative taxonomy of hand grasps.

### Computation of the muscular, kinematic and general quantitative taxonomies: hierarchical super-trees

The capability to merge several highly specific trees is the key idea in obtaining a unique hierarchical structure. This part of the data analysis is fundamental, since it allows us to create a general and global quantitative taxonomy that takes into account inter- and intra-subject variability. Inter-subject variability is due to the highly specific way in which each person performs hand movements. Intra-subject variability is due to the small differences between repetitions of the same movement by the same subject. Despite the inter- and intra- subject variability, each hand movement has common underlying kinematic and muscular patterns that can be extracted by analyzing several repetitions of the same movement performed by different subjects. The variability between subjects can be measured as *edit distance*, that is the minimal-cost sequence of node edit operations that transforms one tree into another [56, 57] (more details in “Supertree similarity measurements” subsection). The common characteristics emerged in a preliminary study [58], where we focused on the taxonomies built on specific subjects (average edit distance of 4.312) and for specific features (average edit distance of 3.548), excluding the generalization phase. More detailed information about these results is reported in Tables 1 and 2, it is worth to notice that we considered the weighted dendrograms for computing edit distances displayed in both tables. Starting from the initial results, in this paper we aim at expanding and enriching the knowledge in the field by merging several features in order to develop a unique and general taxonomy. Considering 40 subjects and 6 repetitions for each subject results in 240 repetitions of each movement, which is a sample large enough to create the taxonomy with the procedure described in this section. The procedure to compute the taxonomies of hand movements starts from the subject-specific hierarchical modality-feature trees and includes several phases. First, subject-independent hierarchical modality-feature trees are computed. Second, the general kinematic and muscular taxonomies of hand movements are computed. Third, a general taxonomy of hand movements is computed.

**Table 1:** Inter-subject variability in grasps across the different quantitative metrics expressed as edit distance

	IAV EMG	MAV EMG	RMS EMG	TD EMG	WL EMG
Muscular	4.60 ± 1.68	4.60 ± 1.68	4.05 ± 1.24	4.36 ± 1.48	4.51 ± 1.60
	IAV glove	MAV glove	RMS glove	TD glove	WL glove
Kinematic	4.04 ± 1.45	4.04 ± 1.45	4.04 ± 1.45	3.95 ± 1.59	3.84 ± 1.28

Rows represent the different modalities while columns represent modality features

**Table 2:** Intra-subject variability in grasps across the different quantitative metrics expressed as edit distance

	Muscular	Kinematic
Subject 1	4.00 ±2.65	1.44 ±1.33
Subject 2	2.48 ±1.75	1.28 ±1.87
Subject 3	0.96 ±1.00	2.40 ±2.59
Subject 4	2.08 ±1.44	0.96 ±1.40
Subject 5	3.04 ±2.05	1.60 ±2.33
Subject 6	3.84 ±2.49	1.44 ±1.65
Subject 7	2.96 ±2.14	1.20 ±1.30
Subject 8	2.24 ±2.03	1.76 ±2.10
Subject 9	4.32 ±2.78	0.64 ±0.93
Subject 10	3.12 ±2.14	1.28 ±1.43
Subject 11	2.80 ±2.10	0.96 ±1.40
Subject 12	0.32 ±0.47	2.24 ±1.99
Subject 13	2.48 ±2.08	1.60 ±1.26
Subject 14	3.12 ±2.07	0.64 ±0.93
Subject 15	2.72 ±1.87	1.92 ±1.74
Subject 16	3.36 ±2.31	1.60 ±1.88
Subject 17	2.16 ±1.67	1.76 ±1.73
Subject 18	2.96 ±1.91	1.28 ±1.87
Subject 19	1.44 ±1.33	0.96 ±1.40
Subject 20	2.32 ±1.64	1.28 ±1.87
Subject 21	2.72 ±1.95	2.00 ±1.81
Subject 22	2.24 ±1.63	2.56 ±2.30
Subject 23	2.32 ±1.59	2.56 ±2.30
Subject 24	1.92 ±1.32	1.68 ±1.49
Subject 25	2.96 ±1.87	0.96 ±1.40
Subject 26	3.52 ±2.28	1.28 ±1.87
Subject 27	2.80 ±1.90	1.04 ±0.96
Subject 28	3.12 ±1.99	0.64 ±0.93
Subject 29	3.28 ±2.41	2.24 ±1.99
Subject 30	3.68 ±2.78	1.60 ±2.33
Subject 31	2.72 ±2.43	2.40 ±2.30
Subject 32	1.84 ±1.51	0.64 ±0.62
Subject 33	3.28 ±2.20	1.04 ±0.96
Subject 34	2.00 ±1.36	1.28 ±1.87
Subject 35	2.48 ±1.75	1.92 ±2.80
Subject 36	2.56 ±2.30	0.96 ±1.40
Subject 37	0.32 ±0.47	1.28 ±1.87
Subject 38	2.56 ±1.70	2.08 ±2.15
Subject 39	2.24 ±1.58	1.28 ±1.87
Subject 40	3.36 ±2.57	2.16 ±2.27

Rows represent subjects while columns represent the different modalities

As said in the previous section, one hierarchical tree is computed for each subject and for each combination of modality-feature, thus leading to five hierarchical trees for the EMG data and five trees for the kinematic data. For each modality and for each feature, a supertree is computed by combining the data of all the 40 subjects, leading to a subject-independent hierarchical modality-feature tree. The procedure used to merge the hierarchical trees is based on the Subtree Prune-and-Regraft (SPR) distance [59]. The calculation of the SPR distance is computationally expensive. Thus, the algorithm combines the Maximum Agreement Forests (MAFs) approach and clustering to make the construction of the SPR-based supertree feasible. Clustering reduces the complexity of the input trees into sub-problems that can be solved recursively. The algorithm solves the MAF problem between a pair of rooted trees by recursively exploring all edge-cutting possibilities. The supertree is built in two phases: the construction of an initial SPR supertree and the SPR rearrangement. The final supertree is a binary rooted tree constructed iteratively by minimizing the SPR distance. This approach was demonstrated to be better than other common distance criteria on biological data sets [59]. Merging the subject-independent hierarchical modality-feature trees of the same modality leads to two modality supertrees: the first one representing the quantitative kinematic taxonomy of hand movements (obtained by merging all the Cyberglove feature trees); the second one representing the quantitative muscular taxonomy of hand movements (obtained by merging all EMG feature trees).

Finally, the quantitative muscular and the kinematic taxonomies of hand movements were merged into the general quantitative taxonomy of hand movements. While the EMG tree gives a vision of muscular involvement in the movement and the kinematic tree shows the actual physiological movement performed by the subject, mixing the two allows a general analysis of the movement from both the muscular and the kinematic perspective.

### Supertree similarity measurements

Evaluating the similarity between the quantitative muscular and the kinematic taxonomies of hand movements can yield fruitful insight, particularly to measure whether the two agree or not. While a reasonable agreement between the two taxonomies may enforce their representativeness, a strong disagreement may lead to a limited acceptability. Intermediate situations can be interesting to highlight differences in the data acquisition modalities or highlight differences between the muscular activation and the actual performed movement. The tree edit distance is frequently used in the comparison of hierarchical trees [56, 57]. The measure is computed as the minimal-cost sequence of node edit operations that transforms one tree into another. The algorithm used to compute the tree edit distance was originally proposed by Pawlik and Augsten [56, 57]. It includes three possible edit operations: delete a node, insert a node and rename the label of a node. A cost was assigned to each edit operation. The cost of an edit sequence is the sum of the costs of its edit operations. The tree edit distance is the sequence with the minimal cost.

## Results

This work presents a quantitative taxonomy of hand grasps based on muscular and kinematic data, described in detail in “General quantitative taxonomy of hand grasps based on muscular and kinematic data” subsection. The general taxonomy is computed by merging the sEMG and kinematic taxonomies of hand grasps (that are described in detail in “Muscular and kinematic taxonomies of hand grasps” subsection) and it is compared in “Comparison with the *GRASP* taxonomy” subsection with a qualitative taxonomy of hand grasps that merges most of previous results described in literature.

### Muscular and kinematic taxonomies of hand grasps

**Fig. 2:**
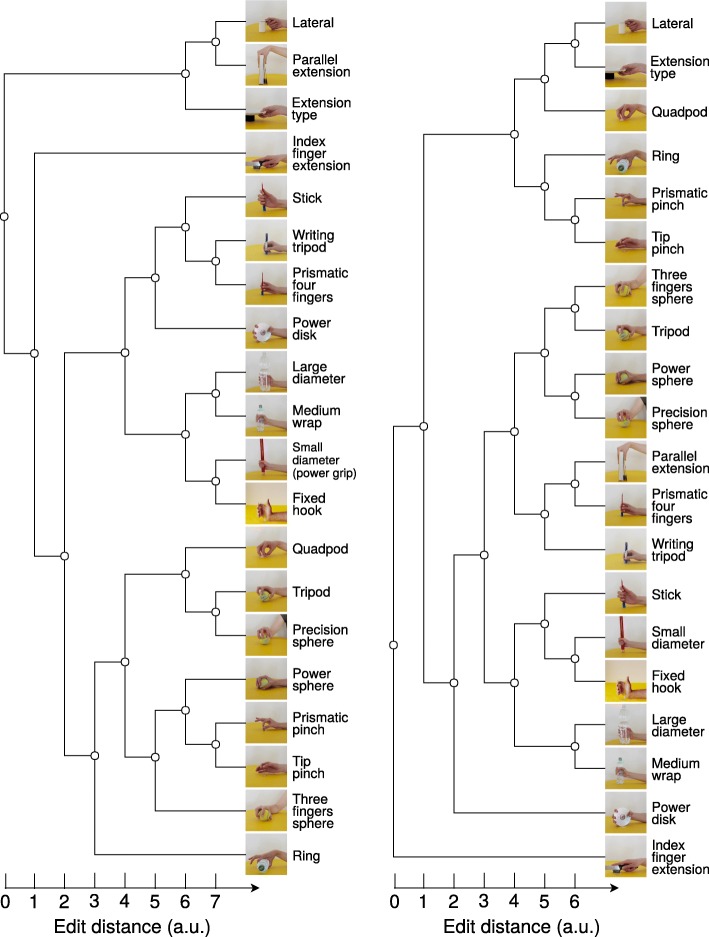
Modality specific quantitative taxonomies of hand grasps. **a** Muscular taxonomy computed from EMG data and (**b**) kinematic taxonomy computed from kinematic data recorded using the CyberGlove. The taxonomies are similar (edit distance = 33)

The quantitative hand movement taxonomies based on EMG (Fig. 2a) and kinematic data (Fig. 2b) are in agreement and provide a similar representation of the hierarchical organization of hand movements. This result is confirmed by the edit distance between the two supertrees, which is 33 (a value within the range of the distances computed for hierarchical trees obtained in a specific modality by using different features). This fact enforces the validity of both taxonomies, that were computed using sensors measuring different parameters related to hand movements. The groups of movements defined in the two modality-specific taxonomies are often similar. For instance, the *large diameter* and *medium wrap* grasps are linked at the first level in both the EMG and the kinematic taxonomy. The same happens for several other groups of movements, such as the *small diameter* and *fixed hook* grasps, the *prismatic pinch* and *tip pinch* grasps. Other movements change from first level connections in one tree to second level connections in the other. This is the case, for instance, of *parallel extension* and the *lateral grasp*, *prismatic four fingers* and *writing tripod*, *precision sphere* and *tripod*. An interesting change happens considering the *power sphere*, *precision sphere*, *tripod* and *three finger* grasps. These grasps are strongly linked (i.e. they are very similar) considering the kinematic taxonomy. In the EMG taxonomy the *precision sphere* and the *tripod* grasps are closer to the *quadpod* movement, while *power sphere* and *three finger* grasps are closer to the *prismatic pinch* and *tip pinch* grasps. Few movements change the grasp group depending on the considered taxonomy. This is the case of the *power disk*, the *index finger extension* and the *parallel extension* grasp. The *power disk* is grouped with the *prismatic four fingers*, the *writing tripod* and the *stick* grasp in the EMG based taxonomy. On the other hand, only in the penultimate level in the kinematic taxonomy is linked to them. The *index finger extension* grasp is isolated in both trees. In the EMG tree, this grasp is in a single branch, close to the majority of graspings but linked at the higher level to the grasp group including the *parallel extension*, *lateral* and the *extension type* grasps. In the CyberGlove tree on the other hand, it is completely isolated from the others movements. A strong difference between the two taxonomies occurs for the *parallel extension* grasp. In the EMG tree, the grasp is isolated from the other movements but grouped with the *lateral* and the *extension type* grasps. In the kinematic taxonomy it is close to the *prismatc four fingers* and the *writing tripod* grasp. There are two possible reasons to explain this difference. First, the EMG taxonomy considers the activation of wrist flexors/extensors, while the taxonomy based on the data glove does not consider them. The EMG signals measure all the muscular activity in the forearm, including the activity related to wrist movements while the Cyber Glove on the other hand is sensitive only to finger movements. Second, the difference can be due to variations in the force used to accomplish the movements, since the EMG signals are sensitive to it. In any case, except these few situations, the differences between the EMG and the kinematic taxonomy of hand movements are limited, confirming the validity of the proposed approaches and thus the validity of both taxonomies.

**Fig. 3:**
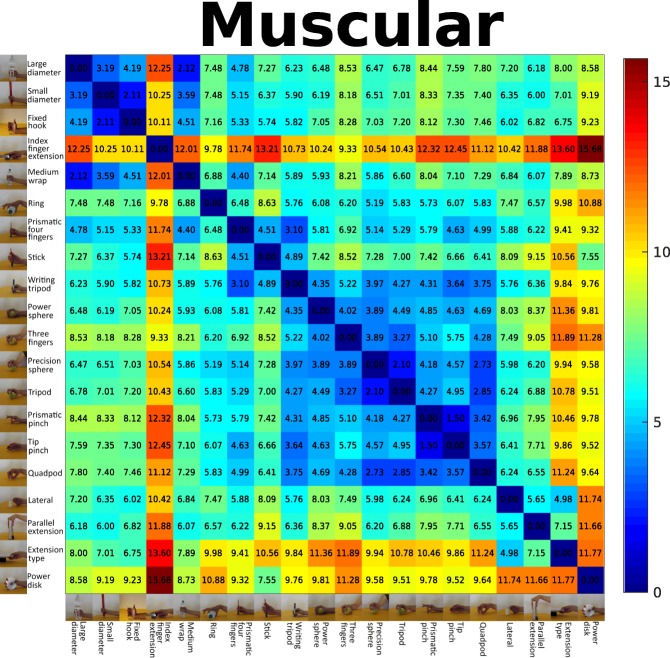
Muscular distance matrix. Distance matrix computed as the mean Mahalanobis distance between the considered grasps, using the available features computed on the EMG data

**Fig. 4:**
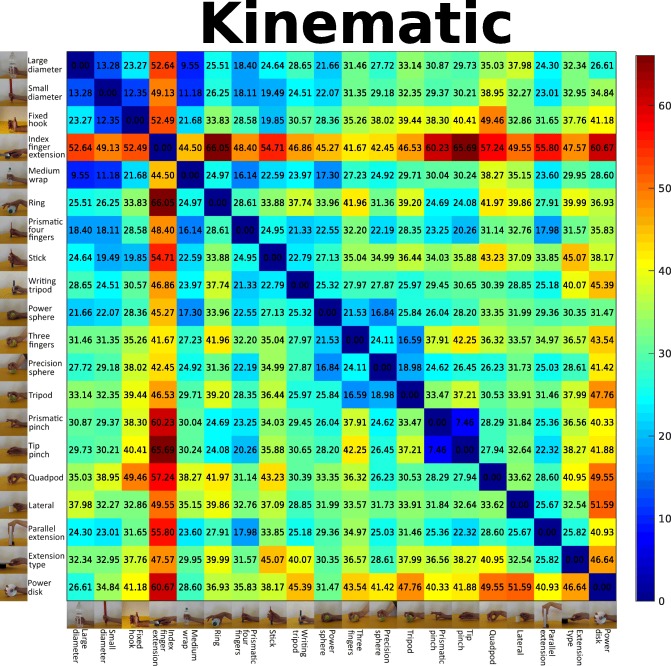
Kinematic distance matrix. Distance matrix computed as the mean Mahalanobis distance between the considered grasps, using the available features computed on the CyberGlove data

As previously said, the final EMG and glove taxonomies are assembled from supertrees built from single features. We measured how much the grasps are similar one to each other by using MANOVA starting from those features. The comparison involved the same features for different movements, so we computed a metric able to cope with the entire distribution in a multi-dimensional space as described in “Hierarchical trees” subsection. Mahalanobis distances were computed for all features, and for each modality, we averaged them to obtain a unique value representing how close a movement is to another. In order to provide an intuitive way to show similarity between EMG and glove information, we built two distance matrices, one for the muscular (Fig. 3) and one for the kinematic (Fig. 4) data. The two matrices show several similarities and, in general, they confirm the considerations derived from the respective supertrees. For example, the *index finger extension* is clearly distant from all the other movements, while *small diameter*, *fixed hook*, *large diameter*, and *medium wrap* are very similar grasps. As a further prove to sustain the idea of merging trees built from different sensors, Table 3 represents the edit distance between the modality-specific taxonomies and the supertrees built on each modality for each specific feature. Considering the EMG data, the IAV and the MAV based taxonomy are the most similar to the muscular taxonomy (edit distance = 22). The edit distance between the IAV and the MAV taxonomies is 0. The most different tree is the one based on WL (edit distance = 39). Considering the kinematic data, the RMS based taxonomy is identical to the kinematic taxonomy (edit distance = 0), while the TD tree is the most different.

**Table 3:** Edit distance between the modality-specific taxonomies of hand grasps and each modality feature supertree

	IAV EMG	MAV EMG	RMS EMG	TD EMG	WL EMG
Muscular Tax.	22	22	34	24	39
	IAV glove	MAV glove	RMS glove	TD glove	WL glove
Kinematic Tax.	19	31	0	34	26

### General quantitative taxonomy of hand grasps based on muscular and kinematic data

**Fig. 5:**
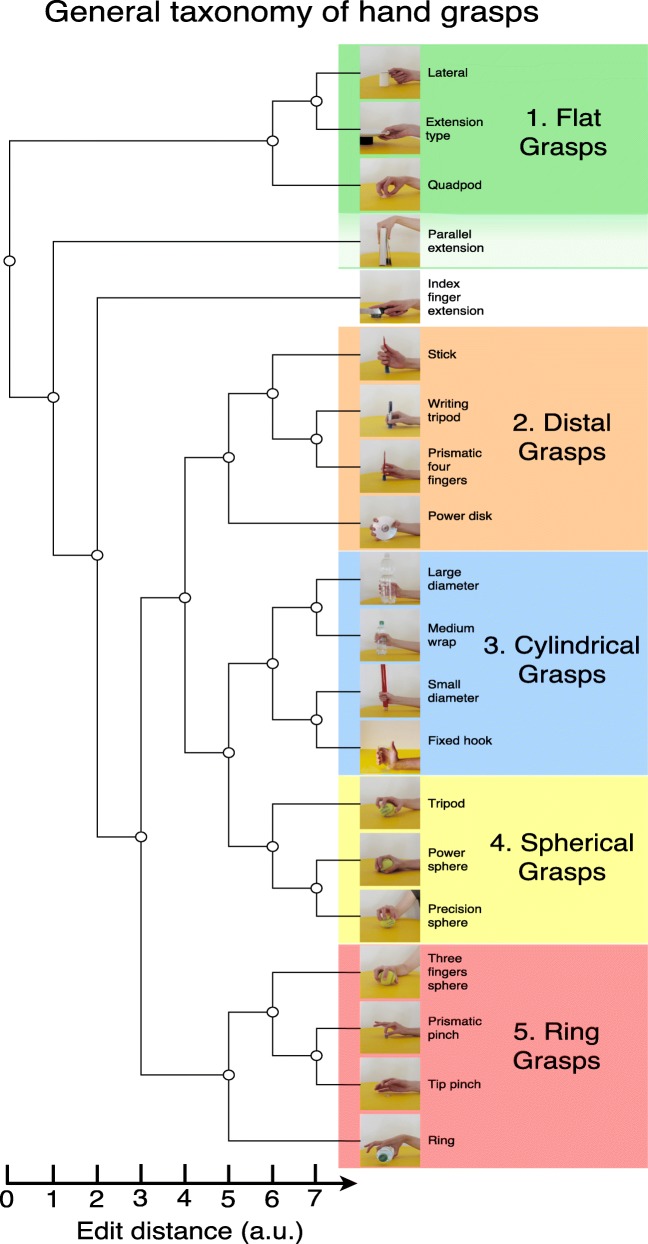
General quantitative taxonomy of hand grasps, obtained by muscular and kinematic data. The parallel extension grasp is not part of group 1 but it may be considered as related to it because of the similarity with the extension grasp and its positioning in the muscular taxonomy

The general quantitative taxonomy of hand grasps (Fig. 5) is computed by merging the muscular and the kinematic taxonomies and offers a general and comprehensive description of hand movement similarities, thus overcoming the subjectivity of previous qualitative taxonomies as well as the limitations of the muscular and the kinematic taxonomies presented in “Muscular and kinematic taxonomies of hand grasps” subsection. The general taxonomy of hand grasps is slightly closer to the EMG taxonomy (edit distance = 29) than to the kinematic taxonomy (edit distance = 42). Coherently, the supersupertree has more connections in common with the EMG one.

The general quantitative taxonomy of hand grasps presents a division into five categories that correspond to real finger positioning and muscular activation, reflecting the shape of the grasped object and balanced combinations of parameters rather than the force used or other specific single parameters. The categories were named as follows according to specific properties of each group: ***1) flat grasps; 2) distal grasps; 3) cylindrical grasps; 4) spherical grasps; 5) ring grasps***. ***Flat grasps*** are well separated from all the others and are characterized by an elongated (or "cupped") positioning of the palm with an abducted or adducted thumb. Parallel extension can be added to this group considering its similarity with the extension grasp and that the grasp is included in the same group in the quantitative muscular taxonomy of hand grasps. ***Distal grasps*** are usually characterized by the strong involvement of distal phalanxes, thus of the flexor digitalis profundus. ***Cylindrical grasps*** are strongly linked to the shape of the object. They usually involve palm opposition with both adducted or abducted thumb and virtual fingers 2-5. ***Spherical grasps*** are strongly linked to the shape of the object as well. They involve both pad and palm opposition with virtual fingers 2-3, 2-4 and 2-5. ***Ring grasps*** are almost entirely in accordance with the *GRASP*’s taxonomy grasps with virtual fingers 2. This category includes as well the *three finger sphere* grasp, which is the only power, pad opposition grasp with virtual fingers 2-3 in the *GRASP* taxonomy. The *three finger sphere* grasp is grouped differently within the muscular and the kinematic taxonomy. These facts suggest that in static conditions the middle finger may have an accessory function in the grasp.

Cylindrical and spherical grasps can also be grouped into a macro-sub group at the third level. Qualitative comparison of the results with the kinematic hand grasping synergies [5] highlights an overall similarity between the cylindrical grasps and the first synergy obtained by Santello et al. (closure of finger aperture achieved by flexion at the pip joints of the fingers and thumb adduction and internal rotation) and between the spherical grasps and the second synergy (flexion at the mcp joint and adduction of the fingers).

The main differences between the general and the muscular taxonomies concern the grasps targeting spherical objects. In the general taxonomy, the *power sphere*, the *precision sphere* and *tripod* grasps are grouped together, similarly to what happens in the kinematic one. The *three finger sphere* is closer to *prismatic pinch*, *tip pinch* and *ring* grasp. Similarly to the kinematic taxonomy, the *extension type*, *lateral* and *quadpod* grasps are grouped separately from all the other movements.

Two more important differences between the general taxonomy and the modality-specific ones are related to two movements that have different connections in EMG and glove trees: *parallel extension* and *index finger extension*. The *parallel extension* grasp is grouped with the *lateral* and *extension* grasp in the EMG taxonomy, while it is grouped with the *prismatic four fingers* and the *writing tripod* grasp in the kinematic taxonomy. In the general taxonomy, the *parallel extension* grasp is located alone in its own branch, separated from almost any other grasp and separated from the *extension*, *lateral* and *quadpod* grasp. The *index finger extension* grasp is represented as completely separated from the others in the kinematic taxonomy. In the EMG and in the general taxonomy of hand movements on the other hand, the grasp is still quite isolated but it is grouped with classical grasps (such as the *stick*, *medium wrap* grasp) at a very high level.

As previously mentioned, the differences between the muscular and the kinematic taxonomies are due to the properties of the movements that the different sensors can highlight. Thus, the general taxonomy of hand grasps provides a unified and general description of all of them.

### Comparison with the *GRASP* taxonomy

Comparing the general quantitative taxonomy of hand grasps with previous taxonomies allows evaluating the considerations used to create the previous taxonomies and to better interpret the results of this paper. Among the taxonomies presented so far, the *GRASP* taxonomy [17] is a a well accepted taxonomy that represents most of the previous studies and includes all the movements considered in this paper. The authors divided the grasps into groups according to four main parameters: 1) power type; 2) opposition type (i.e. the direction in which the hand applies force on the object); 3) thumb position and 4) virtual finger assignments.

**Fig. 6:**
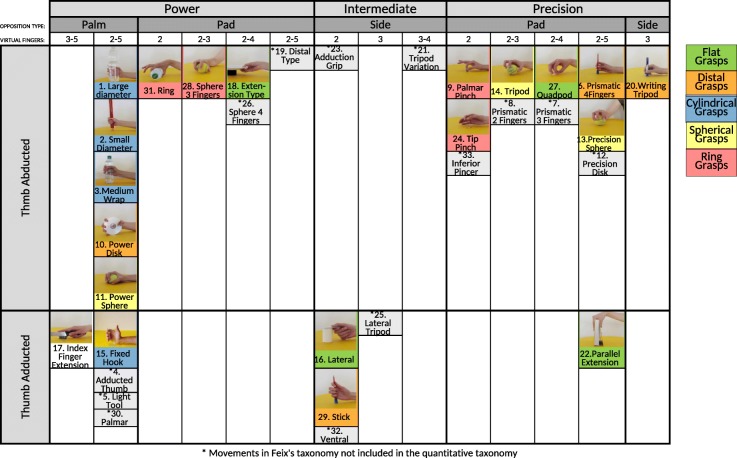
Taxonomy comparison. Comparison of the general quantitative taxonomy of hand grasps with the GRASP taxonomy

The quantitative taxonomy of hand grasps is partially similar to the *GRASP* taxonomy considering the sub-groups determined by the intersection of the *GRASP* parameters. However, it differs in the fact that the parameters considered in the *GRASP* taxonomy are differently (and only partially) represented by the quantitative taxonomy (Fig. 6).

#### *GRASP* taxonomy parameters

The ***subdivision according to power type*** (power, intermediate or precision grasps) that strongly influenced the scientific literature in the past is not well supported by the general and modality-specific quantitative taxonomies of hand movements (power, intermediate and precision grasps are usually divided between the five groups presented in this paper). This result is also confirmed considering only the quantitative muscular and kinematic taxonomies, that are more similar to the general quantitative taxonomy. The ***subdivision according to opposition type*** (pad opposition, palm opposition or side opposition) is partially supported by the general quantitative taxonomy of hand grasps (palm and pad grasps are in most cases well divided). The ***subdivision according to thumb position*** (thumb abducted or adducted) is not well supported by the general taxonomy of hand grasps, even if the *index finger extension grasp* is constantly well separated from all the other grasps and the *lateral* and *parallel extension grasp* are grouped. The ***subdivision according to virtual finger assignments*** is well supported by the general quantitative taxonomy of hand grasps. The *index finger extension grasp* is constantly well separated from the other grasps, coherently with the fact that it is the only grasp with virtual fingers 3-5 in the *GRASP* taxonomy. The general quantitative taxonomy category "ring grasps" is almost entirely in accordance with the *GRASP* taxonomy groups having virtual finger 2. This category includes as well the *three finger sphere* grasp, which is the only power, pad opposition grasp with virtual fingers 2-3 in the *GRASP* taxonomy. Two grasps of the virtual finger 2-4 category in the *GRASP* taxonomy (*extension type* and *quadpod*) are grouped in the category “flat grasps” of the general quantitative taxonomy. However, grasps that are grouped with virtual fingers 3, 2-3 and 2-5 are often mixed within the general quantitative taxonomy of hand grasps.

#### Sub-groups determined by the intersection of the GRASP taxonomy parameters

The ***thumb-abducted, palm opposition, power grasps*** of the *GRASP* taxonomy are grouped together in the general quantitative taxonomy (the only differences are for the *power disk* and the *power sphere*, that are separated in the general quantitative taxonomy). Coherently with the *GRASP* taxonomy, the *fixed hook* is grouped at the second level with the thumb abducted palm opposition power grasps. The *index finger extension* grasp is separated from almost all the others, somehow corresponding to the *GRASP* taxonomy, in which this movement is alone in the power palm thumb adducted group having virtual fingers 3-5.

Coherently with the *GRASP* taxonomy, the *ring* and the *sphere three finger* grasps are grouped in the quantitative taxonomy. However, these grasps are grouped with the *prismatic (palmar) pinch* and the *tip pinch*, which previously were represented as part of the precision grasps. In the *GRASP* taxonomy, the ***thumb-adducted, side opposition, intermediate grasps*** include the *lateral* and *stick* grasps. These two grasps are not grouped in the general quantitative taxonomy of hand grasps. In fact, the *lateral* grasp is grouped with the *extension type* and the *quadpod* grasp. The *stick* grasp is grouped at the second level with the *writing tripod* and the *prismatic four fingers* and, at the third level, with the *power disk*. In the quantitative taxonomy, the ***thumb abduction, precision group*** of the *GRASP* taxonomy is divided into several sub-groups. The sub-groups are often grouped (also with other movements) according to the shape of the object rather than on the properties previously identified. The *prismatic four finger* grasp is grouped with the *writing tripod* at the first level, the *tripod* and the *precision sphere* are grouped at the second level, the *prismatic (palmar) pinch* and the *tip pinch* are grouped at the first level. This result shows that, on average, the shape of the object influences the positioning of the fingers and the muscular activity more than the usefulness for power or precision tasks and opposition type.

Finally, accordance between the general quantitative and the *GRASP* taxonomy is obtained for the *parallel extension* grasp, that is separated from most of the other movements in both of them.

## Discussion

This paper presents to the best of our knowledge the first quantitative taxonomy of hand grasps based on muscular and kinematic measurements of the hand (Fig. 5). Several taxonomies of hand grasps were presented in scientific literature, all of which are based on rigorous qualitative descriptions of hand movements and valuable scientists’ intuitions. Although they are capable of highlighting intrinsically important characteristics of hand movements, a qualitative analysis is prone to subjectivity and it does not allow a demonstrable confirmation or refutation offered by quantitative methods [20]. The quantitative taxonomy presented in this paper is based on experimental measurements and statistical data analyses. The analysis is limited to the considered data and time domain features, that determine the organization of the taxonomy. Further analysis (including other data and signal features, such as for instance frequency based features) is considered in future work. Such analyses may be able to highlight deeper or different relationships between muscular and kinematic properties of hand movements. This paper sets the basis for such work by providing a quantitative data-driven description of the hand movements that are divided into five main groups, as presented in Fig. 5. The general quantitative taxonomy of hand grasps is based on two modality specific taxonomies (based on EMG and kinematic data Fig. 2). The results are interesting both considering the modality-specific and the general quantitative taxonomies.

The modality-specific taxonomies provide very similar representations of the hierarchical organization of hand movements (edit distance = 33), thus validating each other. The similarity between the muscular and the kinematic taxonomy confirms the existence of strong relationships between the muscular activity and the actual motion of the hand, as expected by anatomy. Small differences between the muscular and the kinematic taxonomies exist. Such differences can be due to the differences in the techniques used to record the data. The EMG based taxonomy considers the force exerted and the motion of the wrist, while the taxonomy based on the data glove does not consider these parameters. Nevertheless, these differences are in general small compared to the similarities and they can possibly be reduced by considering weights when merging hierarchical trees, as explained in “Hierarchical trees” subsection. Another possible source of difference can be related to non-linearities existing between some joint angles in the CyberGlove sensor output (e.g. abduction/adduction at the metacarpophalangeal joints) [60, 61]. Although this aspect can affect the kinematic data, the kinematic taxonomies are based on grasp similarities in the kinematic feature space (that take into account the distribution of the data) and not directly the joint angles, probably contributing to the similarity with the muscular taxonomy.

Several parameters can be used to quantitatively characterize hand grasps. This work considers kinematics and muscular activity in order to target the posture of the hands and due to practical data availability. The consistency between the muscular and kinematic taxonomy enforces the usefulness and reliability of the results. The analysis of other parameters can definitely be interesting and should be considered in follow-up work. The edit distance boundaries depend on the number of nodes (thus on the number of considered grasps). Intuitively, the larger the number of classes, the higher the possible number of variations that can occur between different trees. This fact can be one of the reasons behind the discrepancy obtained between the modality trees. Future work should address this fact in detail, by applying additional or alternative operations, measures or approaches.

Depending on the domain, one specific taxonomy may be more useful than the other. While the kinematic taxonomy may be more useful for robotics, the muscular taxonomy may be more suitable for applications in prosthetics. Additionally, both taxonomies can have applications in rehabilitation, physiology and neuroscience. The general taxonomy aims at providing a solution that is intermediate to the different fields, allowing (and hopefully fostering) the collaboration among them on the basis of the first set of quantitative results in this challenging domain. The general quantitative taxonomy provides a comprehensive quantitative representation of hand grasps, overcoming the subjectivity of the taxonomies previously presented in literature and the limitations of the muscular and the kinematic taxonomies presented in this paper. The general quantitative taxonomy suggests a division into five groups of grasps that were named after specific properties of each group: 1) flat grasps; 2) distal grasps; 3) cylindrical grasps; 4) spherical grasps; 5) ring grasps. Cylindric grasps and spherical grasps can also be grouped into a macro-sub group at the third level.

The division in categories is arbitrary, made in order to facilitate the comparison with previous taxonomies and to provide a further synthesis of the taxonomy. Future work could benefit from including quantitative approaches to perform the division in categories.

The comparison of the general quantitative taxonomy of hand movements with previous taxonomies is important because it allows to validate the parameters on which the previous taxonomies were based and to better interpret the results of this paper. The *GRASP* taxonomy [17] represents a proper reference for the comparison because it is one of the most recent qualitative taxonomies of hand grasps and because it is based on the comparison of several previous taxonomies. The quantitative approach only partially confirms the parameters used to create the previous taxonomies (and thus the *GRASP* taxonomy), while it enforces movement groups defined on the basis of real finger positioning and muscular activation, reflecting often the shape of the grasped object and balanced combinations of parameters rather than specific single qualitative parameters. The intersections of different parameters in the *GRASP* taxonomy are partially similar to the general quantitative taxonomy of hand grasps. However, there are still important differences (Fig. 6). Considering each parameter separately, some of the qualitative *GRASP* parameters are not well represented in the quantitative taxonomy and some others are predominant in a few categories. In particular, the subdivision of hand grasps according to power (which strongly influenced the scientific literature in the past), is not well supported by the general quantitative taxonomy of hand movements, while the subdivision into opposition and virtual finger assignments are usually better represented in the general quantitative taxonomy (in particular for specific groups, such as ring grasps). We offer two possible interpretations of these results. First, human intuition and perception enrich previous taxonomies with alternative perceptions of the grasps, such as their usual aim, that is separated from a strictly kinematic or muscular representation of the grasps. Second, it can be important that the parameters of previous taxonomies are considered but it is not easy to balance and weigh the parameters in each movement and category properly only on a qualitative basis.

The hierarchical model of human manipulation and grasping described in this paper improves several fields (including robotics, prosthetics, rehabilitation and physiology) with the quantitative analysis of relationships that were previously widely described in literature on the basis of qualitative parameters. In robotics, the five categories of movements defined in Fig. 5 can help to describe and plan robotic hands according to a clear, solid and simple modular definition of movements. Moreover, the taxonomy can provide a priori information to improve classification algorithms, as proposed in [62]. In prosthetics, the general quantitative taxonomy can foster the development of prosthetic hands that are more suitable for real life situations in terms of both control and mechanical design. For instance, the five categories of movements can be compared with the mechanical properties of the prosthesis, as well as with the ADLs and the movements mostly needed by hand amputees in order to develop modular control systems based on the movement categories. In rehabilitation, the presented taxonomy of hand grasps can improve planning with a better scheduling that prioritizes the categories of movements that are more useful (or more realistically achievable) and thus need to be restored earlier. In recent years, hand synergies gathered importance in physiology, bioengineering, rehabilitation and robotics [5]. Comparing the quantitative taxonomy of hand movements with the hand synergies can highlight relationships between the two. The cylindrical grasps look qualitatively similar to the first kinematic hand grasping synergy obtained by Santello et al. [5], characterized by the closure of the finger aperture achieved by flexion at the pip joints of the fingers and thumb adduction and internal rotation. The spherical grasps look qualitatively similar to the second kinematic hand grasping synergy (flexion at the mcp joint and adduction of the fingers). These considerations provide a coherent relationship between the hand synergies and the quantitative taxonomy approaches.

## Conclusions

In conclusion, this work presents the first quantitative taxonomy of hand grasps based on muscular and kinematic data. The taxonomy clarifies with a solid quantitative approach what was proposed in the field so far based mainly on qualitative assumptions, thus unifying the diverse perspectives presented and offering a scientific reference for the taxonomies of hand grasps. The results were compared with previously presented taxonomies of hand grasps, improving them and clarifying the parameters used to define them. They appear at a first qualitative inspection in accordance with hand synergy studies.

## Abbreviations

ADL: Activity of daily living
BIC: Bayesian information criterion
DNN: Deep neural network
DoFs: Degree of freedoms
EMG: Electromyography
FAI: Finger aperture index
FAIs: Finger aperture indexes
IAV: Integrated absolute value
MAF: Maximum agreement forest
MANOVA: Multivariate analysis of variance
MAV: Mean absolute value
MAVS: Mean absolute value slope
MoCap: Motion capture
MU: Motor unit
MUAP: Motor unit action potential
PCA: Principal component analysis
RMS: Root mean square
sEMG: Surface electromyography
SPR: Subtree prune-and-regraft
SSC: Slope sign changes
TD: Time domain statistics
TSC: Time stamp counter
WL: Waveform length
WT: Wavelet transform
ZC: Zero crossings

## Footnotes

1
http://ninapro.hevs.ch/

2 url: http://www.cyberglovesystems.com/

3 The same mathematical expression is also known as Integrated EMG.
